# Supplementary material for: Real-world efficacy of biological agents in moderate-to-severe plaque psoriasis: An analysis of 75 patients in Taiwan
Source: PLoS One. 2020 Dec 29;15(12):e0244620. doi: 10.1371/journal.pone.0244620 (PMC7771674; doi:10.1371/journal.pone.0244620)
Supplement: Attachment — Submitted filename: Response to the reviewers 1120.doc [file pone.0244620.s001.doc]

Nov 10, 2020

Prof. Joerg Heber

Editor-in-Chief

*PLOS ONE*

Dear Professor Heber,

We are thankful to the reviewers and editors providing constructive critiques and suggestions that helped us to improve the organization and clarity of manuscript. We would like to submit the revision of our manuscript entitled “Real-world efficacy of biological agents in moderate-to-severe plaque psoriasis: an analysis of 75 patients in Taiwan”. Changes have been performed to the manuscript according to the comments from the editor and reviewers and all changes in the text and tables were marked in red. Point-to-point replies to the editor and authors’ comments were listed at the end of this letter. We hope that the revised manuscript would find your approval.

Sincerely,

Chaw-Ning Lee, MD.

Department of Dermatology,

National Cheng Kung University Hospital,

Tainan, Taiwan

Journal requirements:

1. Please ensure that your manuscript meets PLOS ONE's style requirements, including those for file naming. The PLOS ONE style templates can be found at

<https://journals.plos.org/plosone/s/file?id=wjVg/PLOSOne_formatting_sample_main_body.pdf> and

<https://journals.plos.org/plosone/s/file?id=ba62/PLOSOne_formatting_sample_title_authors_affiliations.pdf>

**Reply from authors:** The manuscript was adjusted according to the PLOS ONE's style requirements.

2. Thank you for stating in your ethics statement in the online submission form "No consent was obtained, because the data were analyzed anonymously." Please clarify whether all data were fully anonymized before you accessed them or if the researchers anonymized the data after they obtained the data.

In addition, it appears from your ethics document that the ethics committee waived the requirement for informed consent. If so, please add this statement to your ethics statement.

**Reply from authors:** Thank you for the important comment.

(1) The researchers anonymized the data after obtaining the data.

(2) A relevant statement is added into the ethic statement.

3. Thank you for stating in the text of your manuscript "The study protocol was approved by the Institutional Review Board of National Cheng Kung University Hospital." Please also add this information to your ethics statement in the online submission form.

**Reply from authors:** Thank you for the important comment. The information is added.

4. Please provide a reference for the PASI score.

**Reply from authors:** Thank you for the insightful comment. The information is added.

A reference for the PASI score is added into the manuscript as reference 16 (line 88 and 319).

Langley RG, Ellis CN. Evaluating psoriasis with Psoriasis Area and Severity Index, Psoriasis Global Assessment, and Lattice System Physician's Global Assessment. J Am Acad Dermatol. 51(4):563-9, 2004.

5. Please include the date(s) on which you accessed the databases or records to obtain the data used in your study.

**Reply from authors:** Thank you for the comments. The date is added into the manuscript, “….. Taiwan, and the databases was accessed on July 25, 2019” (line 91).

6. We note you have included a table to which you do not refer in the text of your manuscript. Please ensure that you refer to Table 2 in your text; if accepted, production will need this reference to link the reader to the Table.

**Reply from authors:** Thank you for the comments. The “Table 2” was referred at Line 158 of the text.

**Answers to Review Comments to the Author**

Reviewer 1#

This is a very small sample size with a short follow-up time, so it's difficult to make any conclusions. I think this paper would be a better fit for a psoriasis journal.

**Reply from authors:** We agree that the sample size of this study was relatively small. However, there were several strengths of this work, as stated in the Discussion section. The major strength which made us advantageous to other real world study was that the efficacy of the four biological agents under the standard regimens could be compared in a real-work setting due the reimbursement policy of the National Health Insurance in Taiwan.

Reviewer 2#

The Manuscript “Real-world efficacy of biological agents in moderate-to-severe plaque psoriasis: an analysis of 75 patients in Taiwan” is written in presentable manner. However, the number of patients is very less. Manuscript can be accepted after minor revision, if authors agreed to satisfy following comments.
**Reply from authors:** Thank you for the comments.

Q1. Please clarify annotations “*” and “†” used in table 4 and 3 to avoid confusion. Same sign is also used for author’s equal contribution.

**Reply from authors**: Thank you for the comments. The annotations were adjusted accordingly.

Reviewer 3#

1. Though the article is well written and presented, the sample size could have been large enough.

**Reply from authors**: We agree that the sample size of this study was relatively small. However, there were several strengths of this work, as stated in the Discussion section. The major strength which made us advantageous to other real world study was that the efficacy of the four biological agents under the standard regimens could be compared in a real-work setting due the reimbursement policy of the National Health Insurance in Taiwan.

2. Line no. 88 and 89, authors have written that “the prescription of all biological agents complied with the recommended dose and schedule”.

**Reply from authors**: Thank you for the suggestion. The detail of the dose and schedule is added at line 94-103.

3. The dose and schedule of the dosing should have been specified in the manuscript along with the details of the injections and make.

**Reply from authors**: Thank you for the suggestion. The detail of the dose and schedule is added at line 94-103.

4. Data to support the adverse events cases is inadequate with a short follow-up time.

**Reply from authors**: Thank you for the comments. We agree that the follow-up period was relatively short. As stated in the Discussion, a study with longer follow-up period will be conducted to better reflect the occurrence of AE.

5. However, the discussion part needs to be extensively re-written because there are some major problems with the information presented.

**Reply from authors**: Thank you for the comments. The manuscript has been revised extensively in the text, tables and references.

6. It would be more transparent if the authors provide information on how the authors search and correlate the discussion part.

**Reply from authors**: The references were searched on PubMed by the name of the four biologicals and “real-world study” or “clinical trials”. The discussion part has been revised and updated references are added.

7. I understand that this manuscript is based on clinical studies, however, authors should cite more scientific evidence with proper updated citation.

**Reply from authors**: Thank you for the suggestion. The discussion part has been revised and updated references are added.

8. Since the authors mentioned that the effect of the selected drugs for the treatment of mild-to-moderate psoriasis patients the information there should therefore specific about psoriasis as a combined regimen.

**Reply from authors**: Traditional systemic treatments have to be discontinued within two months after initiation of the biological agents according to the regulation of National Health Insurance in Taiwan. Therefore the effect contributed by concomitant use of traditional biological agents could be neglected. A relevant statement is added into the Method section (Line 101-103).

9. The first half of the conclusion seems irrelevant at some level and main content.

**Reply from authors**: Thank you for the comments. The Conclusion has been revised to better fit the main content of this study.

10. The author must apply some more statistical tools for the confirmation of evidence in the experiment part.

**Reply from authors**: Thank you for the suggestion. A new statistic tool, multivariate generalized estimating equations (GEE) models was used for analysis. Relevant description has been added into the Methods and Results sections (Line 129-135, Line 175-182 and Table 4).
